# Ammonium Hydroxide Enhancement of Dietary Protein in High-Fat Diets Modulates Liver Metabolism Signaling in a Sex- and Age-Dependent Manner in C3H/HeJ Mice

**DOI:** 10.3390/ijms27010403

**Published:** 2025-12-30

**Authors:** Benjamin R. Barr, Indhu Subramaniyan, Li Li, Danielle E. Levitt, Lauren S. Gollahon

**Affiliations:** 1 Department of Biological Sciences, Texas Tech University, 2500 Broadway, Lubbock, TX 79409, USA; benjamin.barr@ttu.edu; 2 Departments of Pharmacy Practice and Pharmaceutical Sciences, North Texas Clinical Pharmacology Cancer Core, Texas Tech University Health Sciences Center, 5920 Forest Park Ln, Dallas, TX 75235, USA; indhumathy.subramaniyan@ttuhsc.edu (I.S.); li.li@ttuhsc.edu (L.L.); 3 Department of Kinesiology and Sport Management, Texas Tech University, 2500 Broadway, Lubbock, TX 79409, USA; danielle.levitt@ttu.edu

**Keywords:** dietary protein, ammonium hydroxide enhancement, MASLD, MASH, HCC, diet-induced obesity, metabolites, dietary intervention

## Abstract

(1) Lifestyle changes to modify unhealthy dietary patterns with the goal of preventing MASLD have proven challenging. Here, dietary proteins and their modification with ammonium hydroxide enhancement (AHE) provide molecular evidence that this novel approach may attenuate the development of MASLD without undue dietary adjustments, potentially bypassing non-compliance. (2) High-fat diets containing dietary beef (HFB) or casein (HFC) + AHE (HFBN and HFCN, respectively) were fed to 256 C3H/HeJ female and male mice long term. At 6, 12, or 18 months, hepatic samples were analyzed with targeted metabolomics (glucose, lactate, alanine, glutamine, carnitine) and Western analysis (β-catenin, glutamine synthetase, CYP3A4). RNA sequencing was performed on samples collected at 18 months (*n* = 3; male HFC *n* = 2). (3) Metabolomics results showed that at 18 months, hepatic glutamine was greater in HFBN versus HFCN in females, whereas in males, hepatic glutamine, glucose and lactate were lower in HFBN versus HFCN. Additionally, diets with AHE decreased β-catenin and CYP3A4 protein expression in males. Ingenuity pathway analysis (IPA) of RNA-seq data predicted that HFBN activates PPARα signaling in the liver in both sexes compared to HFCN. Inflammatory activity showed predicted activation for females in the HFBN:HFCN comparison. In males, the inflammatory pathway molecular mechanisms of cancer was predicted as deactivated in HFBN:HFCN. (4) Dietary protein source impacts outcomes, and these outcomes improved with AHE. The HFBN diet improves signaling associated with lipid utilization for females and males, and improved inflammatory signaling for males compared with HFCN. Further exploration of AHE as a dietary intervention in high-fat diets is warranted.

## 1. Introduction

Metabolic dysfunction-associated steatotic liver disease (MASLD) has been designated as the most common cause of chronic liver disease [1]. MASLD is characterized by the accumulation of lipid within the liver in association with systemic metabolic dysfunction [2]. As lipids accumulate, the disease progresses into metabolic dysfunction-associated steatohepatitis (MASH), further into cirrhosis, and/or potentially hepatocellular carcinoma (HCC) [2]. Key in the transition from MASLD to MASH is an increase in hepatic inflammation connected with lipid accumulation [2]. Additionally, during the progression to MASH, inflammation promotes fibrosis resulting in further damage to the liver, including cirrhosis and HCC development over time [3]. Although the major characteristic of MASLD is lipid accumulation in the liver, the exact mechanisms regulating this accumulation and the methods for its treatment remain to be discovered [2]. Currently, disease progression is mainly linked to diet-induced obesity (DIO) and poor dietary habits, including overconsumption of high-fat (HF) red meats [4,5,6]. Furthermore, there are genetic, environmental, and sex-linked factors that may contribute to MASLD development [7]. There are few pharmacological interventions for MASLD [3]. Rather, treatment is focused on weight loss through surgery, lifestyle modification, and more recently, GLP-1 receptor agonists and other anti-obesity drugs [3]. In more severe cases that have progressed to MASH or cirrhosis, liver transplants are the only viable treatment option [1,8]. Arguably, the most beneficial and cost-effective treatment is to modify lifestyle patterns, particularly in the form of dietary intervention [3,9,10]. Such interventions typically require transitioning from a Westernized diet (e.g., overconsumption of alcohol, sugars, processed meats, etc.), to a healthier alternative like a Mediterranean-style diet [11]. Dietary guidelines for Americans have been published by the USDA and USDHHS since 2014, recommending Dietary Approaches to Stop Hypertension (DASH) [12]. Multiple studies have shown that the DASH diet improves overall metabolic health and can attenuate or reverse the progression of liver disease more effectively than a standard “healthy diet” [13,14]. However, regardless of dietary recommendations, adherence to lifestyle interventions remains the primary barrier for most individuals [15,16].

In 2020, food as medicine was introduced as an approach for treatment or prevention of the obesity epidemic as well as other diseases [17]. Currently, food as medicine is limited to only whole foods and their natural components or extracts [18,19,20,21,22]. However, there are also certain food processing techniques designed with the intent to improve health outcomes. Recently, one such processing method, the enhancement of dietary protein sources (DPS) with ammonium hydroxide (AHE), has demonstrated potential benefits for metabolic health and liver metabolism under DIO conditions [23,24]. Results showed that diets with AHE for 12 weeks improved glucose clearance in male and female mice [23]. A long-term study showed AHE- and DPS-dependent differences in lean and fat mass in both male and female C3H/HeJ (C3H) mice fed HF diets, modeling chronic DIO [24]. In the same study, the hepatic lipid droplet (LD) perimeter was smaller in females fed HF casein (HFC) + AHE (HFCN) diets than the group fed untreated HFC for 12 months [24]. In contrast, males fed HF beef (HFB) + AHE (HFBN) had smaller LDs than those fed untreated HFB for 12 months [24]. The major impact from these studies was the potential of AHE and DPS to be used as long-term dietary interventions. However, little is understood about the molecular mechanisms through which different dietary proteins and AHE may benefit metabolic health [24].

**Figure 1:**
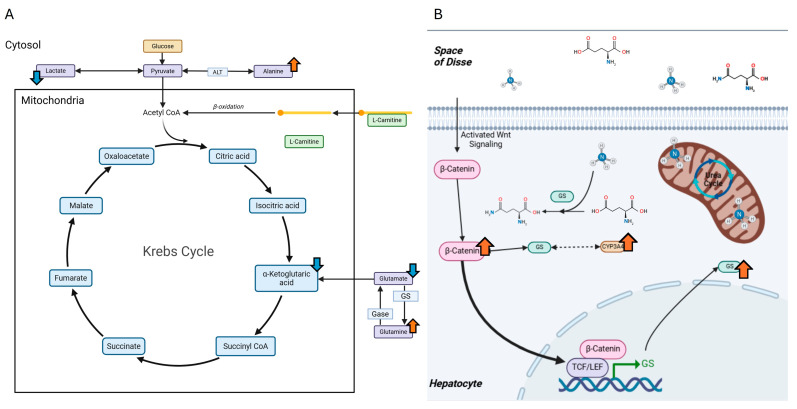
Predicted AHE-induced changes to hepatic metabolite levels and proteins associated with ammonia metabolism. (**A**) A simplified representation of the Krebs cycle. Arrows indicate predicted levels of metabolites that may be affected by AHE. (**B**) The role of β-catenin in the typical high-affinity low-capacity clearance of ammonia in hepatocytes utilizing GS. Additionally, CYP3A4 is predicted to change, through a yet undetermined mechanism potentially associated with GS (dashed, double-headed arrow). Blue arrows = predicted decreases. Orange arrows = predicted increases.

In conjunction with results from prior studies, the current study aims to identify potential molecular mechanisms through which AHE may improve liver health under DIO in female and male C3H mice. Additionally, to determine if a diet containing AHE proteins can modify expression of β-catenin, GS, or CYP3A4 in DIO conditions, a multi-faceted molecular approach was used to analyze the liver transcriptome of mice following 18 months of HFD with casein or beef proteins ± AHE, as well as targeted metabolite and protein levels after 6, 12, and 18 months. Based on their role as potential contributors to the ammonia metabolism and detoxification, the levels of glutamine, glutamate, alanine, carnitine, and glucose were measured using liquid chromatography mass spectrometry (LC-MS) and assessed for AHE- and DPS-associated changes [25]. Predicted AHE-induced changes in these metabolites and their role in ammonia metabolism and clearance through glutamine are shown in Figure 1A. Moreover, the protein levels of β-catenin, glutamine synthetase (GS), and cytochrome p450 3A4 (CYP3A4) were measured because they are key in clearing ammonia, particularly in pericentral hepatocytes [26,27]. One previously published study found that both GS and CYP3A4 were upregulated in hepatocyte-like cells that were generated using ammonia selection [28]. While an increase in GS was expected, increase in CYP3A4 was unexpected, and although both proteins are typically expressed in zone III hepatocytes (pericentral), CYP3A4 functions mostly in drug metabolism and clearance [28]. Additionally, GS expression is regulated by β-catenin signaling in the Wnt pathway [27]. In a study by Dai et al., GS knockout was shown to accelerate HCC development, which was contrary to previous expectations [27]. In another study, when β-catenin is knocked out in mice, the expression of some CYP family members was lost, while others remained unaffected [26]. CYP3A4 is not directly regulated β-catenin [29,30]. However, it has been demonstrated that activation of the Wnt/β-catenin pathways inhibits PPARα-mediated transcription of the CYP3A4 gene [30]. Additionally, in a study from Sekine et al., GS expression was also lost as a result of β-catenin knockout [26]. The canonical roles of β-catenin, GS, and CYP3A4 are shown in Figure 1B. Arrows indicate predicted changes due to AHE of DPS. Outcomes from this study provide evidence for innovative dietary approaches to treating MASLD that may have better rates of adherence compared to traditional dietary modification recommendations.

## 2. Results

### 2.1. Canonical Pathways (CP) and Diet-Induced Differences

Using Ingenuity Pathway Analysis (IPA) software version 01-23-01 (Qiaqen), comparisons in changes to CPs in liver sections collected at 18 months were made between diet groups which shared at least one experimental variable (DPS or AHE) within each sex (see Section 4.3 for comparisons). Z scores indicate the likelihood for the predicted changes to each CP based on previously published information available across a large database.

#### 2.1.1. Predicted Changes to Canonical Signaling Pathways in Females

**Figure 2:**
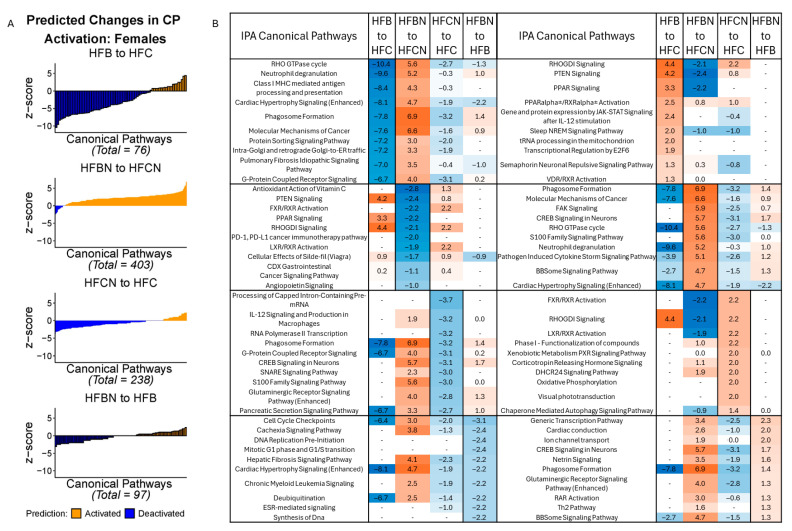
IPA predictions of activation or deactivation of canonical pathways (CPs) in female mice dietary comparisons. (**A**) Predicted activation status for CPs in females based on *z* score from IPA at 18 months. Blue indicates predicted deactivation and orange indicates predicted activation. In comparisons containing < 100 CPs, black outlines have been used to indicate individual CPs. (**B**) Predictions for the top 10 activated and deactivated CPs in females by dietary comparison at 18 months. HFC = HF casein, HFCN = HF casein + AHE, HFB = HF beef, and HFBN = HF beef + AHE. The complete tables as well as a breakdown of the top 20 pathways can be found in the Supplemental File S1.

In the females, the highest number of CPs predicted to be changed was observed in the HFBN:HFCN comparison (403; majority activated). HFB:HFC had the fewest predicted changes (76; majority deactivated). Figure 2A summarizes the predicted changes in activation status for CPs in each of the dietary comparisons by *z* score. Additionally, *z* scores for the predictions of the top 10 activated and deactivated CPs for each comparison (HFB:HFC, HFBN:HFCN, HFCN:HFC, and HFBN:HFB) are shown in Figure 2B. Each column in Figure 2B includes the predicted status of the other pairs for comparison. Notable changes in activation status for the CPs identified between HFB:HFC and HFBN:HFCN were: molecular mechanisms of cancer, neutrophil degranulation, PPAR signaling, and PPARα/RXRα activation. In the HFB:HFC comparison, molecular mechanisms of cancer [−7.6 (*z* score)] and neutrophil degranulation [−9.6] were predicted to be deactivated, while PPAR signaling [3.3] and PPARα/RXRα activation [2.5] were predicted to be activated. The opposite response was observed in the HFBN:HFCN comparison where molecular mechanisms of cancer [6.4] and neutrophil degranulation [5.2] were predicted to be activated, with PPAR signaling [−2.2] deactivation predicted. Notably, PPARα/RXRα activation [0.82] was predicted to be activated in HFBN:HFCN. Results for the comparison of HFCN:HFC predicted deactivation of glutaminergic receptor signaling pathway (enhanced) [−2.8], while activation [1.3] was predicted in HFBN:HFB. Z scores for these and all other identified pathways for each comparison can be found in Supplementary File S1: IPA Table from Females.

#### 2.1.2. Predicted Changes to Canonical Signaling Pathways in Males

**Figure 3:**
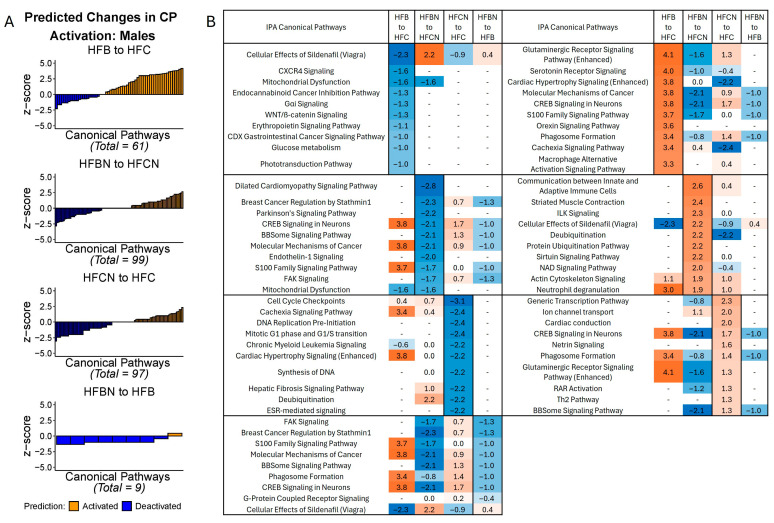
IPA predictions of activation or deactivation of canonical pathways (CPs) in male mice dietary comparisons. (**A**) Predicted activation status for CPs in males based on *z* score from IPA at 18 months. Blue indicates predicted deactivation and orange indicates predicted activation. In comparisons containing < 100 CPs, black outlines have been used to indicate individual CPs. (**B**) Predictions for the top 10 activated and deactivated CPs in males by dietary comparison at 18 months. HFC = HF casein, HFCN = HF casein + AHE, HFB = HF beef, and HFBN = HF beef + AHE. The complete tables as well as a breakdown of the top 20 pathways can be found in the Supplemental File S2.

In the males, the greatest number of CPs predicted to be changed was observed in the HFBN:HFCN comparison (99 identified; majority deactivated). HFBN:HFB showed the fewest predicted changes (9; majority deactivated). Figure 3A summarizes the predicted changes in activation status for CPs in each of the dietary comparisons in males, by *z* score. Additionally, the *z* scores for the predictions of the top 10 activated and deactivated CPs for HFB:HFC, HFBN:HFCN, HFCN:HFC, and HFBN:HFB comparisons are shown in Figure 3B. Each subtable in Figure 3B indicates the predicted status of the other pairs for comparison. Notable changes in activation status in the HFB:HFC and the HFBN:HFCN comparisons were molecular mechanisms of cancer and glutaminergic receptor signaling pathway (enhanced). In the HFB:HFC comparison, molecular mechanisms of cancer [3.8], and glutaminergic receptor signaling pathway (enhanced) [4.1] were predicted to be activated, while these CPs were predicted to be deactivated [−2.1, and −1.6, respectively] in the HFBN:HFCN comparison. Additionally, neutrophil degranulation was predicted to be activated in both the HFB:HFC and HFBN:HFCN comparisons [3 and 1.9, respectively], while mitochondrial dysfunction was predicted to be deactivated for both comparisons [−1.6 and −1.6, respectively]. The last notable change is in the HFBN:HFB comparison for which deactivation of molecular mechanisms of cancer [−1] was predicted. Additionally, Regulation of Lipid Metabolism by PPARα was predicted to be activated in HFBN:HFCN [*z* score 1.3, Supplemental File S2]. Z scores for these and all other identified pathways for each comparison can be found in Supplementary File S2: IPA Table from Males.

### 2.2. Changes to Liver Metabolite Levels

**Figure 4:**
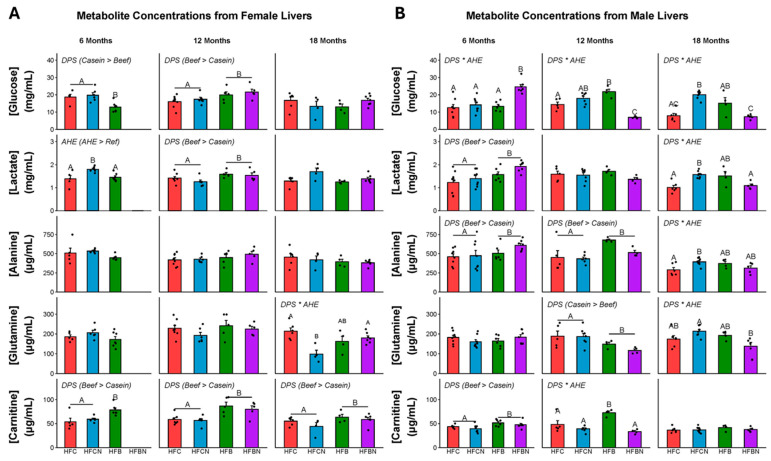
Levels of metabolites identified in female and male C3H/HeJ livers after 6, 12, and 18 months for each diet. Data are presented as mean ± SEM. Significance between diet groups is indicated at *p*-value < 0.05 by different letters (i.e., result A is significantly different from result B or result C; AB denotes no significance from results A or B; AC denotes no significance from results A or C). Red indicates HFC, blue indicates HFCN, green indicates HFB, and purple indicates HFBN. (**Panel A**) Metabolite concentrations at 6, 12, and 18 months in females. (**Panel B**) Metabolite concentrations at 6, 12, and 18 months in males. Glucose and lactate are measured in mg/mL, while alanine, glutamine and carnitine are measured in μg/mL. Note: HFBN data for 6-month-old females is not available.

Metabolites were extracted from the livers of all diet groups at three timepoints (6, 12, and 18 months) to generate a sex-specific metabolomic profile for each diet. Targeted metabolites were glucose, lactate, glutamine, alanine, carnitine, glutamic acid, citric acid, and succinic acid. Measures of glutamic acid, citric acid, and succinic acid demonstrated large within-group variance and are not presented here. F-statistics and *p*-values for the main effects of AHE and DPS and their interactions can be found in Table S1. For females, concentrations of each metabolite at each timepoint can be found in Figure 4A. At 6 months in females, a significant main effect for DPS was observed for glucose (casein > beef), AHE for lactate (AHE > ref), and DPS for carnitine (beef > casein). At 12 months, there were significant main effects of DPS for glucose, lactate, and carnitine (beef > casein). At 18 months, a significant DPS × AHE interaction effect was observed for glutamine, where glutamine was elevated in the HFC and HFBN diets compared with HFCN. Also at 18 months, a significant main effect of DPS was observed for carnitine (beef > casein).

**Figure 5:**
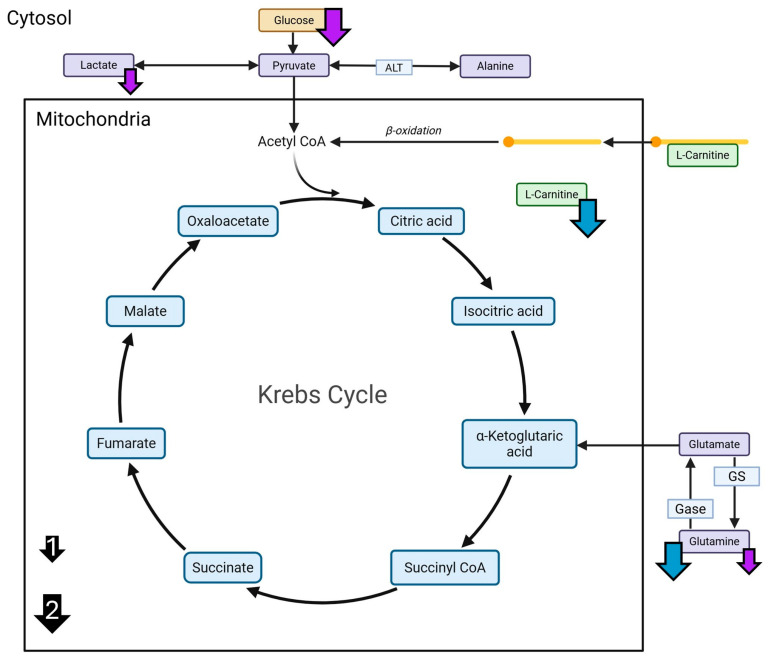
A summary of AHE-induced changes in targeted liver metabolites. Measured changes in targeted metabolites for the HFCN females and HFBN males at 18 months. Glucose, alanine, lactate, carnitine, and glutamine were measured using LC-MS. Blue arrows indicate decreases in liver metabolites from HFCN females at 18 months compared with two other diets. Purple arrows indicate decreases in liver metabolites from HFBN males at 18 months compared one or two other diets. Arrow size indicates number of other diets, with the smaller arrows (1) indicating one diet and larger arrows (2) indicating two diets (shown in black).

For males, concentrations of each metabolite at each timepoint can be found in Figure 4B. F-statistics and *p*-values for the main effects of AHE and DPS and their interactions can be found in Table S2. At 6 months in males, a significant interaction of DPS × AHE was observed for glucose. Glucose was elevated in HFBN-fed males compared with all other groups. Also at 6 months, there were significant main effects of DPS for lactate, alanine, and carnitine (beef > casein). At 12 months, significant DPS × AHE interaction effects were observed for glucose and carnitine. Glucose was significantly lower in the HFBN than the other diet groups and carnitine was elevated in the HFB compared with the other diet groups. Main effects for DPS were observed for alanine (beef > casein) and glutamine (casein > beef). At 18 months, significant DPS × AHE interaction effects were found for glucose, lactate, alanine, and glutamine, with HFCN having the highest levels of each. Glucose was greater in the HFCN compared to the HFC and HFBN diets, and the HFB diet has greater glucose than the HFBN. HFCN has greater lactate compared with the HFC and HFBN. HFCN has greater alanine than the HFC and greater glutamine than the HFBN. A summary of AHE-induced changes in targeted liver metabolites is shown in Figure 5.

### 2.3. Levels of Key Proteins Linked with Ammonia Metabolism

**Figure 6:**
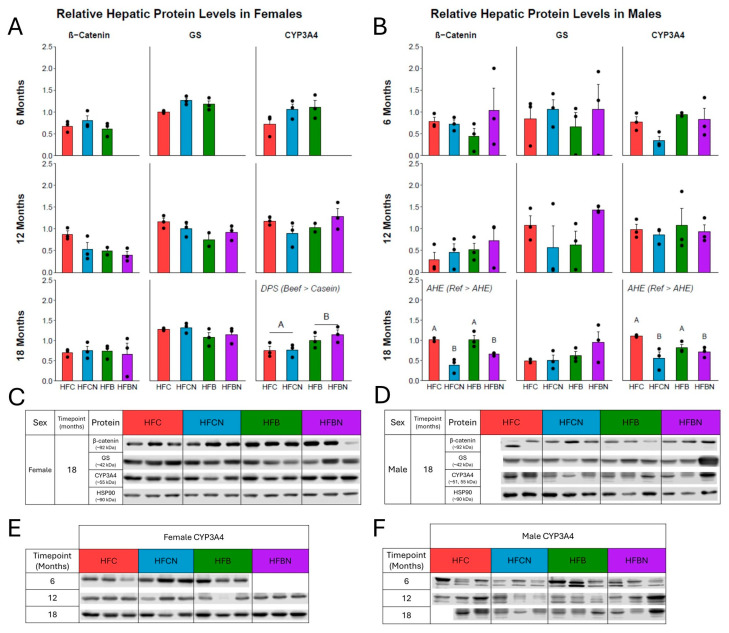
Densitometric measures of relative expression of key proteins linked with ammonia metabolism. (**A**) Female relative expression as mean ± SEM at 6, 12 and 18 months, respectively. (**B**) Male relative expression as mean ± SEM at 6, 12, and 18 months, respectively. (**C**) Representative Western blots of each protein at 18 months in females. (**D**) Representative Western blots of each protein at 18 months in males. Proteins analyzed: top: β-catenin (mw ~90 kDa, ProteinTech, 1:10,000), second: GS (mw ~42 kDa, NovusBio, 1:1000), and third: CYP3A4 (mw ~51–55 kDa, ProteinTech, 1:10,000). Bottom: HSP90 (mw ~90 kDa) used as the reference. (**E**,**F**) CYP3A4 Western blots from each timepoint for females and males, respectively. Significance between diet groups is indicated at *p*-value < 0.05 by different letters (i.e., result A is significantly different from result B). Samples were not available for assessment in HFBN females at 6 months; the third replicate in HFB females at 12 months was not available due to an issue with protein lysate stability; the third replicate for HFC males at 18 months was not available due to lack of non-cancerous liver tissue for lysis.

Relative expression of proteins measured in livers from females at 6, 12, and 18 months are displayed in Figure 6A and from males for the same timepoints in Figure 6B. *p*-values and F-statistics can be found in Tables S3 and S4 for females and males, respectively. In female livers at 18 months, there was a significant main effect of DPS for CYP3A4 expression (beef > casein) (Figure 6A). At 18 months, there were significant main effects of AHE for β-catenin and CYP3A4 expression in male livers (ref > AHE) (Figure 6B). No interaction between DPS and AHE was identified for β-catenin, GS, or CYP3A4 from livers from either sex at any timepoint. Representative immunoblot images of β-catenin, GS, CYP3A4, and HSP90 (housekeeping) in livers from each sex at 18 months are presented in Figure 6C,D. Additionally, to highlight unique time- and sex-specific CYP3A4 expression patterns, the immunoblot images for all three timepoints are presented in Figure 6E,F. However, since protein expression analyses were not powered for statistical comparison across timepoints or between sexes, further in-depth exploration is outside the scope of this study.

## 3. Discussion

The present findings indicate that DPS alone, and with AHE, alters the hepatic transcriptional profile together with markers of glucose, lipid, and ammonia metabolism in an age- and sex-specific manner. The following discussion will examine the influence of AHE and DPS on selected physiological functions first in females and then in males. The IPA results aligned strongly with our central hypothesis. Although they are predictions, they also denote important relationships and future areas of research that will be validated with orthogonal assays.

### 3.1. Females

#### 3.1.1. MASLD Progression in Female Mice

A CP modulated by DPS on a background of AHE is PPAR Signaling. PPAR Signaling was predicted to be deactivated in the HFBN:HFCN comparison and activated in the HFB:HFC comparison. Notably, PPARα/RXRα Activation was predicted to be activated in both comparisons (HFB:HFC and HFBN:HFCN). This likely indicates that while other PPARs are deactivated in HFBN:HFCN, PPARα is activated. PPARα signaling has a negative correlation with the progression of MASLD towards MASH. However, in healthy individuals, hepatic PPARα levels are highest in the acute fasting state when whole-body energy requirements are being met primarily by lipid stores [31,32]. Based on findings from other studies, it appears that in the chronically fed (or overfed) state, PPARα signaling is decreased as hepatic lipids accumulate, restricting the ability of the liver to utilize excess lipids for energy (β-oxidation), intracellular signaling, or lipoprotein formation (LDL, VLDL, etc.) [33,34]. Further, early MASLD progresses towards MASH in the chronically overfed state, where PPARα signaling appears to be further reduced [33,34]. It has been demonstrated by other studies that PPARα is a CYP3A4 promoter [35,36]. Previously, we reported that the HFBN and HFCN females had distinctly different body compositions, with greater total and fat mass observed in the HFCN females at 18 months [24]. At 18 months, the relative level of CYP3A4 in the HFBN diet is elevated compared with the HFCN diet. The predicted activation of PPARα/RXRα Activation for the HFBN:HFCN comparison reflects the elevated levels of CYP3A4 in the HFBN diet. In conjunction with this, it indicates that the HFBN females had higher capacity for β-oxidation, potentially due to increased PPARα signaling or stabilization of PPARα by USP25, than the HFCN females. It was demonstrated in January 2025 that ubiquitin-specific peptidase 25 (USP25), a deubiquitinating enzyme, is capable of directly increasing PPARα and, in turn, decreasing HF diet-induced hepatic lipid accumulation [37]. As we previously reported, LD size in the HFCN females was larger than in the HFBN, further emphasizing that DPS on an AHE background modulates MASLD progression towards MASH [24].

Although energy metabolism is difficult to directly examine using the current data, with the high levels of lipid sequestering and the predicted change to PPARα/RXRα Activation, it is also important to further consider β-oxidation. Fatty acid oxidation, or β-oxidation, is the process of preparing long chain fatty acids (FA) for utilization as acetyl-CoA within the Krebs cycle. This occurs using carnitine to shuttle FAs across mitochondrial membranes (outer and inner) into the mitochondrial matrix. A major source of dietary carnitine comes from red meat (mammalian skeletal muscle) and whole milk protein sources [38,39]. While it can be found in other dietary protein sources, it is typically in much lower levels [38,39]. However, casein is a milk protein isolate, which means that during its isolation, carnitine and other protein components like whey are removed in its formulation [6,38,39]. Thus, in the present study, we anticipated that carnitine levels would be elevated in the diets containing beef compared with diets containing casein, and this is observed in the females at all timepoints. A previous study identified that supplementation with polyunsaturated fatty acids (PUFAs), polyphenols, and carnitine as well as carnitine alone increased levels of PPARα transcripts in HepG2 cells measured with rt-PCR [40]. This further supports predicted activation of PPARα in the HFBN:HFCN comparison. Future studies should plan to directly examine the levels of PPARα mRNA as well as PPARα protein at all timepoints to confirm the dietary influences on this specific pathway. While further investigation into the state of PPARα and β-oxidation is required for confirmation, it is likely that in females, HFBN diets attenuate the progression of MASLD phenotypes compared with HFC and HFCN diets through activation of PPARα signaling.

#### 3.1.2. Inflammatory Signaling and Cancer for Female Mice

Hepatic glutaminergic receptor signaling (enhanced) was predicted to be activated in female mice fed HFBN compared with HFCN and HFB, indicating another potential modification by DPS on a background of AHE. This signaling pathway is usually associated with neuronal synapses which heavily rely upon excitatory amino acid transporters (EAATs) (QIAGEN Inc., Hilden, Germany, https://digitalinsights.qiagen.com/IPA, accessed on 27 February 2025), and these same EAATs are expressed in hepatic stellate cells (HSCs) [41]. Predicted activation of glutaminergic receptor signaling from the liver tissue in the current study is most likely from HSCs. When activated, these cells function to rebuild the extracellular matrix in the case of liver injury, while continued activation leads to hepatic fibrosis [42]. Similar to neurons, HSCs heavily utilize EAATs, as well as glutamate recycling enzymes like GS or glutamate dehydrogenase (GDH) [43]. In the present study, no differences were observed between diets in levels of GS at any of the timepoints for females. Despite this finding, it is possible that levels of other proteins associated with the activation of HSCs (e.g., GDH or specific EAATs) could be altered by DPS or AHE, and this should be examined in future studies. Recently, it was demonstrated that elevated glutamine (GLN) activates HSCs, and this HSC activation was strongly correlated with fibrosis progression in alcohol-associated liver disease (ALD) [43]. A subsequent study examining natural killer (NK) cell function revealed that elevated extracellular GLN functions to recruit NK cells to the liver for the removal of activated HSCs [44]. The present study demonstrates higher levels of hepatic glutamine in livers from HFBN-fed than HFCN-fed females at 18 months, supporting the predicted activation of glutaminergic receptor signaling (enhanced) from the HFBN:HFCN comparison.

Hepatic lipid accumulation, particularly the formation of supersized LDs identified in MASLD and MASH [45], is associated with inflammation and cancer [46,47,48]. Interestingly, our previous study identified larger LDs in the HFCN diet than in the HFBN diet, although generally the average perimeters for both groups would be considered “giant” (or “supersized”) LDs [24,45]. Typically, larger LDs are associated with liver disease progression towards inflammation and fibrosis [49,50]. In the current study, neutrophil degranulation and molecular mechanisms of cancer have different predicted activation states as the result of DPS on a background of AHE. In HFB:HFC, these CPs were predicted to be deactivated, while activation was predicted in HFBN:HFCN. The IPA description of neutrophil degranulation describes that activation leads to the recruitment of neutrophils to inflammatory sites, followed by the release of granular vesicles essential to the innate immune response (QIAGEN Inc., https://digitalinsights.qiagen.com/IPA, accessed on 27 February 2025) [41]. Chronic activation of this pathway may serve as a potential molecular mechanism increasing the likelihood of cancer incidence, as the links between inflammation and cancer have been extensively explored [51]. Notably, our previous study reports low incidence of HCC across all diets for females [24], likely indicating that the activation of the neutrophil degranulation CP predicts acute, rather than chronic, inflammatory signaling. Additionally, the current study found that levels of β-catenin and its downstream product GS, did not significantly differ between diets, indicating canonical Wnt signaling [27,52]. This supports acute activation of neutrophils, as elevated levels of β-catenin would indicate hyperactivation of the Wnt/β-catenin signaling pathway which has been classically linked to the progression of cancer, including HCC [27].

### 3.2. Males

#### 3.2.1. MASLD Progression in Male Mice

In males, regulation of lipid metabolism by PPARα [z score 1.3, Supplemental File S2] is predicted to be activated in the HFBN:HFCN. As a promoter of CYP3A4 [35,36], PPARα activation should result in higher levels of CYP3A4 in HFBN compared with HFCN. However, in the present study, differences in levels of hepatic CYP3A4 were only identified between the AHE diets compared with the reference diets at the 18-month timepoint (expression was lower in HFCN and HFBN compared with HFC and HFB). A potential explanation for the difference in predicted activation state for the regulation of lipid metabolism by PPARα with similar levels of CYP3A4 would be the pattern of hepatic lipid accumulation observed over the course of the study. Our previous findings indicate that average LD size for the HFCN only slightly increased (~55 µm to ~60 µm perimeter) between 12 and 18 months, while the average perimeter for the HFBN went from ~35 µm to ~85 µm in the same timespan [24]. Notably, the number of LDs appeared fewer in the HFBN than in the HFCN, although LD counts were not reported [24]. This change in hepatic lipid accumulation reflects a delayed onset of MASLD in HFBN, and likely a different point in the PPARα signaling timeline associated with lipid utilization and storage in an overfed state compared with HFCN. Additionally, it is possible that alternative signaling pathways associated with lipid metabolism not identified in the present RNA-seq analysis, and which do not promote CYP3A4 in the AHE diets compared with the reference diets, may be involved. One example of this would be activation of PPARγ with reduced PPARα signaling [53]. It was demonstrated that in PPARα knockout mice fed an HF diet, PPARγ mRNA was upregulated in the liver along with some of the genes typically promoted by PPARα associated with lipid metabolism (e.g., CYP4A10 and CD36) [53,54]. However, currently there are no studies reporting a link between the activation of PPARγ and the promotion of CYP3A4 [55]. Additionally, changes in carnitine levels for males are mainly observed at 6 and 12 months. In males, hepatic carnitine is elevated in the HFB diets ± AHE over the casein diets ± AHE at 6 months. At 12 months, carnitine is only elevated in the HFB diet compared with all other diets (HFBN, HFC and HFCN). Then at 18 months, carnitine is no longer different between diets even though the amount of dietary carnitine available with beef DPS should be greater than in the casein DPS, as previously discussed [6]. Based on the critical role of carnitine in lipid metabolism, the decrease observed in HFB-fed males at 12 months is surprising, and it indicates a potential bioenergetic shift away from FA metabolism. Our previous study reported relatively consistent average LD perimeter (~50–60 µm) between 12 and 18 months for the HFB males; however, survivability for this group was drastically lower than the others in the same time frame [24]. This limits the examination of LDs in the previous study and carnitine in the present study to only viable individuals at this timepoint [24]. Further exploration of DPS and AHE is required in the context of hepatic lipid storage and metabolism; however, the HFBN appears promising to delay the onset of MASLD in males. Directions for future investigation should include examination of HSC status and function in the AHE diets, PPARα and PPARγ signaling, and carnitine utilization with respect to a chronic dietary pattern over time and in aging for males.

#### 3.2.2. Inflammatory Signaling and Cancer for Male Mice

Glucose, lactate, and glutamine were all reduced in the HFBN diet compared with the HFCN diet at 18 months in the present study. Additionally, glutaminergic receptor signaling was predicted to be deactivated in the HFBN:HFCN comparison indicating a reduction in hepatic fibrosis signaling that was supported by lower glutamine levels [43]. Neutrophil degranulation was predicted to be modified for the males mainly by DPS, with activation being predicted in both beef diets when compared with their casein counterparts (HFB:HFC and HFBN:HFCN). Interestingly, for males, molecular mechanisms of cancer were predicted to be deactivated for HFBN:HFCN, but activated for HFB:HFC. This could be explained by acute inflammation within the HFBN compared with the HFCN group, while the HFB compared with the HFC likely indicates a chronically inflamed state associated with metabolic dysfunction. Alternatively, predicted activation in the HFBN:HFCN could be an indication of other components within molecular mechanisms of cancer not associated with inflammation. Regardless, further exploration of inflammatory status is required [56]. In addition to the predicted status of molecular mechanisms of cancer, relative β-catenin levels are lower in diets with AHE (HFBN and HFCN). Canonically, β-catenin levels are positively associated with GS levels [27]. However, in the present study, there was no difference in GS levels across diets at 18 months. Lower levels of β-catenin in the AHE diets may be caused by non-canonical negative regulation of β-catenin by GS. Recently, along with the positive relationship between β-catenin signaling and GS (canonical), a negative feedback component was identified where GS competes for N-cadherin binding sites with β-catenin (non-canonical) [57]. This ultimately results in degradation of β-catenin through ubiquitination [57]. Although this molecular interaction was identified in gastric cancers, GS has also been identified to limit HCC progression through maintenance of nitrogen metabolism and suppression of mTORC1 [27]. In the present study, lower levels of glutamine were measured in the HFBN males compared with the HFCN males at 18 months, although levels of GS were not different. This indicates that GS is potentially being utilized in the non-canonical manner, negatively regulating β-catenin signaling through competition for N-cadherin binding sites [27,57]. Liver tumor incidence from our previous study supports this hypothesis as HFCN-fed males had lower incidence of HCC than HFC-fed males [24]. Notably, the HCC incidence in the HFBN-fed males (highest survivorship) was slightly lower than in HFCN, but poor survivorship in the HFB-fed males resulted in the lowest HCC incidence (HCC incidence: HFC > HFCN > HFBN > HFB) [24]. Future studies including examination of hepatic inflammatory status, zonal contribution to GS levels along with glutamine (and other amino acid) concentrations, and evaluation of potential negative regulation of β-catenin by GS will be essential in elucidating the roles of DPS and AHE in progression of MASLD to HCC in males.

## 4. Materials and Methods

### 4.1. Animal Study, Diets, and Liver Tissue Storage

The C3H/HeJ (C3H) mouse strain serves as an alternative model for studying obesity development, particularly in non-predisposed populations over time. Animal handling followed regulatory compliance under the approved Texas Tech University IACUC protocol number 19021-02 (Approval date: 12 February 2019). A total of 256 female (128) and male (128) C3H mice were obtained at 4 weeks old from Jackson Laboratories (Bar Harbor, ME, USA) and allowed 1 week to acclimate. Mice were housed four per cage under controlled temperature and humidity and in 12 h light/dark cycle rooms. Each sex was randomly split into four (32 mice per diet per sex) HF diets (46% kcal fat): casein (HFC), casein with AHE (HFCN), beef (HFB), and beef with AHE (HFBN). Diets and water were supplied ad libitum for the duration of the study. Complete dietary formulations can be found in our previous publications, as well as findings indicating that the HFC dietary group developed diet-induced obesity (DIO) over the course of the study [24,58]. Each week, diet consumption per cage and individual mouse mass was recorded, in addition to a visual assessment of health. At cross-sectional time points of 6, 12, and 18 months, ~8 mice (up to 8, all available based on adverse events) were sacrificed from each sex and diet group via CO_2_ euthanasia followed by cervical separation. Unscheduled euthanasia was performed as per protocol for co-morbidity endpoints described in [24,58]. Autopsies were performed on these mice and tissues were collected. For scheduled collection timepoints, liver tissues were collected at 6, 12, and 18 months and were bisected. Half of each liver was flash frozen in liquid nitrogen while the other was stored in 4% paraformaldehyde, as previously described [24]. Flash frozen liver tissue was used for all analyses in the present report.

### 4.2. RNA Extraction and Isolation from Cancer-Free Livers for RNA-Seq

RNA was isolated from healthy livers from (18-month-old) female and male C3H/HeJ mice for RNA-seq to determine alterations to signaling pathways and identify molecular changes associated with the development and progression of MASLD. Sample size for liver RNA extraction was *n* = 3/sex/group, except for in the male HFC (*n* = 2) as healthy sampling was limited by formation of liver tumors. Random tumor-free sections (~30 mg) of flash-frozen whole livers were dry homogenized using the BioSpec Mini-BeadBeater 96, and solid aluminum vial rack (BioSpec, Bartlesville, OK, USA). The aluminum vial rack was frozen in liquid nitrogen before the 2 mL tubes containing each liver section and 2 mm homogenizing beads were added. Tissues were then homogenized for 3 min at 2000 rpm. Tissue homogenates were suspended in lysis buffer (Buffer RLT) from the Qiagen RNeasy Mini Kit. After obtaining suspended tissue lysates, RNA extraction was performed using the Qiagen RNeasy Mini Kit as per manufacturer’s instructions, including RNA Cleanup and On-Column DNase Digestion (Qiagen, Hilden, Germany). RNA yield and purity were measured utilizing the NanoDrop^®^ ND-1000 Spectrophotometer (Thermo Scientific, Waltham, MA, USA).

### 4.3. Ingenuity Pathways Analysis (IPA)

**Figure 7:**
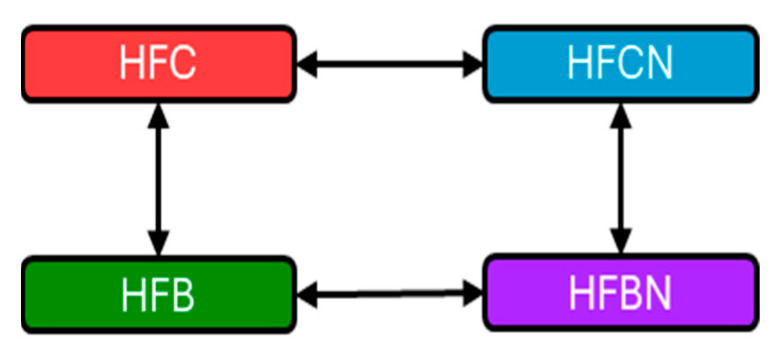
Graphical representation of comparisons made within IPA. Red = HFC, blue = HFCN, green = HFB, and purple = HFBN. Double headed arrows indicate comparisons. Note: Casein is considered the reference dietary protein for beef.

RNA-seq was performed after stranded library prep using an Illumina NovaSeq 6000 (Illumina, San Diego, CA, USA) with S1 flow cell at 25 million reads per sample. RNA-Seq data were obtained by the Texas Tech Center for Biotechnology and Genomics as .fastq files from the extracted total mRNA. Files were uploaded and mapped to the Mus musculus reference genome in ArraySTAR^®^ (ArrayStar INC Rockville, MD, USA). Biological replicates were grouped (typically *n* = 3) and reads per kilobase per million mapped reads (RPKM) were compared for both females and males separately. Four comparisons were made for each sex, with non-AHE or casein protein as references (see Figure 7). Then, genes with ≥2 fold-change at 95% confidence interval (Student’s *t* test, no correction) as calculated automatically by ArrayStar were exported to a data table for each comparison. Exported data tables included experimental *p*-value (as calculated previously), RPKM for the experimental diet (e.g., HFBN), RPKM for the reference diet (e.g., HFB), and the experimentally determined fold-change in expression of each gene. Additionally, annotations of gene names were included in the exported data file. Comparison files were then uploaded to IPA. Each comparison was subjected to IPA’s core analysis to generate lists of modulated canonical signaling pathways (CPs), among other analyses, as well as a list of expressed genes measured from RNA-seq used in the core analysis to generate predictions. The activation status based on Z score for the identified CPs was then displayed using ggplot (v3.5.1). 

### 4.4. Targeted Metabolomics

Tumor-free sections (~30 mg) of flash-frozen livers were sent to the North Texas Clinical Pharmacology Cancer Core (NTCPCC, Texas Tech University Health Sciences Center, Dallas, TX, USA) for targeted metabolomics. Tissues were lysed in Pierce IP lysis buffer with protease inhibitor (1 Roche cocktail tablet per 10 ml buffer) at a tissue-to-buffer ratio of 10 mg:50 µL [25]. The mixture was then homogenized using a mechanical probe in a 2.0 mL centrifuge tube for 30 s at 10,000 rpm. From this, 50 µL of the homogenate was extracted for metabolite quantification. To precipitate proteins, 450 µL of methanol was added to the 50 µL aliquot and vortexed for 30 s. Precipitate containing tissue debris was separated via centrifugation at 4000 rpm at 4 °C for 10 min. The supernatant was then stored at −20 °C until analysis. Standard curves were generated at a range of concentrations (0.1, 0.5, 1, 2.5, 5, 10, 50, 100, 500 and 1000 µg/mL (glucose); 0.01, 0.05, 0.1, 0.5, 1, 2.5, 5, 10, 50 and 100 µg/mL (lactate); or 0.5, 1, 5, 10, 50, 100, 500, 1000 and 2000 ng/mL (alanine, glutamine, L-carnitine)), depending on the targeted metabolite. Standards and samples were run on LC-MS. The complete details of LC-MS methods have been previously published [25]. Samples for each sex, diet and timepoint reflect the number of mice that survived until sacrifice and had healthy (i.e., tumor-free) liver tissue available. Sample sizes (*n* = 4–8) can be found in Table 1.

### 4.5. Protein Extraction and Quantification

To extract proteins, a random section (~50 mg) of each flash-frozen whole liver (68 in total) was added to 700 μL of lysis buffer (Pierce RIPA Lysis and Extraction Buffer Thermo Scientific, product #PI89900) with Halt Protease and Phosphatase Inhibitor Cocktail 100× (Thermo Scientific, product #PI78440) with 2 mm homogenizing beads and placed on ice. Tissues were homogenized using the VWR^®^ Bead Mill Homogenizer (6 m/s, 1 min) in sets of 12 (Avantor, Allentown, PA, USA). After homogenization, lysates were centrifuged for 20 min at 4 °C. Supernatant was separated and centrifuged for an additional 5 min at 4 °C, placed in a fresh tube, and any remaining precipitates were discarded. Following this, the Pierce BCA Protein Assay (Thermo Scientific, product #PI23227) was used to determine the protein concentration from each sample. BSA standard concentrations were prepared according to the manufacturer’s instructions; 200 μL of working reagent was added to 25 μL of each BSA standard and liver protein sample in triplicate. Reference (BSA) and sample protein concentrations were quantified at 562 nm on the Bio-Tek Synergy H1 Microplate Reader H1M (Agilent, Santa Clara, CA, USA).

### 4.6. Immunoblotting

Concentrations of extracted protein were normalized with RIPA and then an equal amount of 6× Laemmli dye (5% β-mercaptoethanol) was added to each sample for a final protein concentration of 2.5 μg/μL. Samples were then denatured by heating to 95 °C for 5 min and placed on ice. A measured 25 μg of sample (10 µL of prepared sample) was loaded into each well of hand-cast SDS-polyacrylamide gel (4% stacking and 10% resolving). The reference ladder used was PageRuler^TM^ Plus Prestained Protein Ladder (Thermo Scientific, product #26619). Protein was separated on the gel at 120 V until 10 kDa ladder proteins reached the bottom of the gel (~90 min). The wet transfer technique was then used to transfer protein from the gel to nitrocellulose membranes (Thermo Scientific, product #88018, Lot YA3443897). Transfer was performed at 4 °C in pre-chilled 1× transfer buffer (25 mM Tris; 192 mM glycine, 20% MeOH) with an ice pack at 90 V for 90 min. After, Ponceau S solution (0.1% Ponceau S, in 5% glacial acetic acid) was used to assess protein transfer. Immediately after this, membranes were blocked with 5% BSA in 1× TBST (0.1% tween-20) for one hour. After blocking, membranes were incubated with primary antibody in 5% BSA in TBST. Primary antibodies and their incubation conditions were: β-catenin at RT for 1 h (1:10,000; ProteinTech, Rosemont, IL, USA: 66379-1-Ig), GS at 4 °C overnight (1:1000; NovusBio, Centennial, CO, USA: NB110-41404), CYP3A4 at 4 °C overnight (1:10,000; ProteinTech, Rosemont, IL, USA: 67110-1-Ig) and HSP90 (housekeeping) at RT for 1 h (1:1000; Cell Signaling Technology, Danvers, MA, USA: C45G5). The order of protein detection was β-catenin and GS first, followed by HSP90 and CPY3A4, respectively, after one strip. After incubation with primary antibodies, membranes were washed 3 times in 1× TBST for 10 min each. Then, membranes were incubated with the appropriate secondary antibody, Goat Anti-Rabbit IgG HRP (1:1000, Cell Signaling Technology: 7074) or Goat Anti-Mouse IgG HRP (1:10,000, Abcam, Waltham, MA, USA: ab6728) for 1 h at RT. Membranes were washed 3 times with 1× TBST for 10 min each before being exposed to ECL (SuperSignal^TM^ West Pico PLUS Chemiluminescent Substrate, Thermo Scientific, Waltham, MA, USA) for 5 min. Images were then developed and visualized using the Odyssey XF Licor Imaging system. Following detection by ECL, membranes were stripped as appropriate using Restore PLUS Western Blot Stripping Buffer (Thermo Scientific, Waltham, MA, USA, product #46430 Lot: F392796) for 5 min and rinsed once with 1× TBST. Images were analyzed in FIJI (FIJI 2.14.0/1.54g/Java 1.8.0_322) to determine density of each protein band. Densitometry results were compared against HSP90 as the loading control.

### 4.7. Statistical Analysis

Statistical analyses for metabolomic and protein data were performed in R. All the R scripts, the raw data, and the Supplementary Files can be accessed through the following github link at https://github.com/BenjaminBarr/Lipid_Metabolism_Signaling/releases/tag/IJMS, (accessed on 27 February 2025). Normality was assessed using QQ-plots of the modeled residuals. Removal of outliers resulted in acceptable normality for each model. Additionally, each model provided robust handling of outliers and thus all outliers were ultimately included in the final analyses. For metabolites, two-way ANOVA was performed via lme4 (v1.1-35.3) with fixed effects for DPS and AHE. Each comparison was performed within timepoints and sexes [59]. For relative protein levels, significance was assessed using nested (technical replicates of each biological sample) two-way ANOVA with fixed effects for DPS and AHE using lme4 (v1.1-35.3). Since HSP90 expression appeared to vary between timepoints and sexes, statistical comparisons were conducted within timepoint and sex. Comparisons for metabolites and proteins in females at 6 months were made using two one-way ANOVAs for each fixed effect (DPS or AHE) due to the absence of samples from females in the HFBN group at 6 months. For each of the ANOVA analyses, Tukey’s HSD post hoc analysis was performed where appropriate using emmeans (v1.10.2). Results are displayed as mean ± SEM with significance considered at *p* < 0.05.

## Figures and Tables

**Table 1:** Sample size for metabolite analysis by sex, diet, and timepoint.

		Time Point		
Sex	Diet	6 Months	12 Months	18 Months
Females	HFC	5	8	6
	HFCN	6	6	4
	HFB	7	5	4
	HFBN	-	5	7
Males	HFC	8	5	6
	HFCN	8	7	8
	HFB	6	4	4
	HFBN	7	4	5

## Data Availability

All the R scripts, the raw data, and the Supplementary Files can be accessed through the following github link at https://github.com/BenjaminBarr/Lipid_Metabolism_Signaling/releases/tag/IJMS, (accessed on 24 December 2025). The original contributions presented in this study are included in the article/Supplementary Materials. Further inquiries can be directed to the corresponding author.

## Supplementary Materials

The supporting information can be downloaded at: https://www.mdpi.com/article/10.3390/ijms27010403/s1.
