# Supplementary material for: Ammonium Hydroxide Enhancement of Dietary Protein in High-Fat Diets Modulates Liver Metabolism Signaling in a Sex- and Age-Dependent Manner in C3H/HeJ Mice
Source: Int J Mol Sci. 2025 Dec 30;27(1):403. doi: 10.3390/ijms27010403 (PMC12787167; doi:10.3390/ijms27010403)
Supplement: Supplementary file 1 [file ijms-27-00403-s001.zip › IJMS_Supplementary.pdf]

Table S1 – ANOVA Table for Metabolite Concentrations in Females

1-way ANOVA for AHE or DPS at 6-Months				
	Statistic	AHE	DPS	AHE x DPS
Glucose	*p*	0.04	<0.01	--
	F (1, 16)	4.89	17.16	--
Lactate	*p*	<0.01	0.23	--
	F (1, 16)	13.75	1.53	--
Alanine	*p*	0.15	0.06	--
	F (1, 16)	2.33	3.94	--
Glutamine	*p*	0.09	0.13	--
	F (1, 16)	3.21	2.50	--
Carnitine	*p*	0.26	<0.01	--
	F (1, 16)	1.39	15.28	--
2-way ANOVA for AHE x DPS at 12-Months				
	Statistic	AHE	DPS	AHE x DPS
Glucose	*p*	0.24	0.01	0.93
	F (1, 20)	1.47	7.46	0.01
Lactate	*p*	0.24	0.02	0.53
	F (1, 20)	0.40	0.15	0.59
Alanine	*p*	0.75	2.21	0.30
	F (1, 20)	0.16	0.24	0.59
Glutamine	*p*	2.15	1.46	0.30
	F (1, 20)	5.71	1.43	15.08
Carnitine	*p*	0.67	<0.01	0.68
	F (1, 20)	0.18	23.14	0.18
2-way ANOVA for AHE x DPS at 18-Months				
	Statistic	AHE	DPS	AHE x DPS
Glucose	*p*	0.87	1.00	0.08
	F (1, 17)	0.03	0.00	3.56
Lactate	*p*	0.02	0.07	0.15
	F (1, 17)	6.71	3.89	2.33
Alanine	*p*	0.33	0.20	0.76
	F (1, 17)	1.00	1.78	0.09
Glutamine	*p*	0.02	0.35	0.00
	F (1, 17)	6.25	0.94	14.36
Carnitine	*p*	0.33	0.04	0.55
	F (1, 17)	1.03	4.93	0.37

*Highlighted cells indicate those with p-values < 0.05.

Table S2 – ANOVA Table for Metabolite Concentrations in Males

2-way ANOVA for AHE x DPS at 6-Months				
	Statistic	AHE	DPS	AHE x DPS
Glucose	*p*	<0.01	<0.01	<0.01
	F (1, 25)	15.87	14.18	9.765354
Lactate	*p*	0.06	<0.01	0.49511
	F (1, 25)	3.88	10.33	0.479323
Alanine	*p*	0.25	0.07	0.367916
	F (1, 25)	1.40	3.51	0.840849
Glutamine	*p*	0.77	0.75	0.093483
	F (1, 25)	0.09	0.10	3.040792
Carnitine	*p*	0.13	<0.01	0.936528
	F (1, 25)	2.41	11.26	0.006471
2-way ANOVA for AHE x DPS at 12-Months				
	Statistic	AHE	DPS	AHE x DPS
Glucose	*p*	0.01	0.09	<0.01
	F (1, 16)	8.295875	3.352206	48.05
Lactate	*p*	0.16	0.75	0.20
	F (1, 16)	2.13	0.11	1.76
Alanine	*p*	0.09	0.01	0.16
	F (1, 16)	3.35	9.05	2.18
Glutamine	*p*	0.59	0.01	0.43
	F (1, 16)	0.31	9.02	0.64
Carnitine	*p*	<0.01	0.10	0.01
	F (1, 16)	21.75	3.04	10.32
2-way ANOVA for AHE x DPS at 18-Months				
	Statistic	AHE	DPS	AHE x DPS
Glucose	*p*	0.01	0.01	<0.01
	F (1, 19)	9.21	7.54	46.60
Lactate	*p*	0.08	0.62	<0.01
	F (1, 19)	3.46	0.25	25.69
Alanine	*p*	0.14	0.73	0.01
	F (1, 19)	2.36	0.12	9.37
Glutamine	*p*	0.84	0.03	<0.01
	F (1, 19)	0.04	5.43	10.18
Carnitine	*p*	0.57	0.31	0.37
	F (1, 19)	0.34	1.09	0.84

*Highlighted cells indicate those with p-values < 0.05.

Table S3 – ANOVA Table for Relative Protein Expression in Females

		β-catenin			Glutamine Synthetase			CYP3A4		
Age (Months)	Statistic	AHE	DPS	AHE x DPS	AHE	DPS	AHE x DPS	AHE	DPS	AHE x DPS
6	*p*	0.19	0.32	--	0.09	0.69	--	0.49	0.30	--
	F (1,7)	2.07	1.16	--	4.01	0.17	--	0.53	1.24	--
12	*p*	0.05	0.07	0.30	0.72	0.07	0.17	0.83	0.34	0.10
	F (1,7)	5.30	4.57	1.26	0.13	4.63	2.40	0.05	1.07	3.49
18	*p*	0.93	0.87	0.71	0.53	0.08	0.89	0.46	0.02	0.52
	F (1,8)	0.01	0.03	0.15	0.42	4.01	0.02	0.60	9.23	0.46

*Highlighted cells indicate those with p-values < 0.05.

Table S4 – ANOVA Table for Relative Protein Expression in Males

		β-catenin			Glutamine Synthetase			CYP3A4		
Age (Months)	Statistic	AHE	DPS	AHE x DPS	AHE	DPS	AHE x DPS	AHE	DPS	AHE x DPS
6	*p*	0.36	0.96	0.28	0.44	0.83	0.81	0.17	0.08	0.40
	F (1,8)	0.93	0.00	1.37	0.66	0.05	0.06	2.29	4.05	0.81
12	*p*	0.42	0.29	0.93	0.66	0.53	0.07	0.58	0.73	0.96
	F (1,8)	0.72	1.29	0.01	0.20	0.44	4.39	0.34	0.13	0.00
18	*p*	<0.01	0.13	0.16	0.40	0.13	0.42	0.03	0.70	0.08
	F (1,7)	31.61	2.93	2.42	0.81	2.89	0.74	8.01	0.16	4.21

*Highlighted cells indicate those with p-values < 0.05.
